# Case Report: Recurrent vestibular episodes in a dog resembling Meniere’s disease

**DOI:** 10.3389/fvets.2026.1913317

**Published:** 2026-08-25

**Authors:** Maximilian E. Ladenburger, Katarina Kuzek, Annika K. Schmitz, Andreas Brühschwein, Andrea Fischer

**Affiliations:** Small Animal Clinic, Center for Clinical Veterinary Medicine, Ludwig-Maximilians-Universität München, Munich, Germany

**Keywords:** auditory brainstem response, canine, deafness, hearing impairment, nystagmus, otoacoustic emissions, vertigo, vestibular syndrome

## Abstract

Ménière’s disease, characterized by episodic vertigo, sensorineural hearing loss, and tinnitus, is well-documented in humans but rarely reported in veterinary medicine. This case report describes a 15-year-old male mixed-breed dog with recurrent vestibular episodes lasting 30 min to several hours. Clinical signs included ataxia, head tilt, and nystagmus. MRI and diagnostic workups ruled out common structural causes of vestibular signs and ear diseases. Audiometric testing revealed bilateral sensorineural hearing loss with preserved high-frequency hearing in the right ear, a pattern resembling that seen in human Ménière’s disease. While the dog improved with supportive care, multiple relapses occurred until the age of 18. This case suggests a Ménière’s-like syndrome in dogs and highlights the need for hearing function assessments in idiopathic vestibular disease.

## 1 Introduction

Ménière’s disease (MD) is a well-documented disorder in human medicine, dating back to its first description by Prosper Ménière in 1861 (1). According to current clinical practice guidelines, the diagnosis of definite MD relies on specific clinical criteria (2). These include two or more spontaneous episodes of vertigo, each lasting between 20 min and 12 h, accompanied by fluctuating aural symptoms (hearing loss, tinnitus, or aural fullness) in the affected ear. Audiometry must confirm a low- to mid-frequency sensorineural hearing loss during or after an episode, distinguishing MD from age-related presbycusis, which characteristically affects higher frequencies. Furthermore, the diagnosis mandates the strict exclusion of other peripheral or central vestibular disorders. Cases presenting with the typical clinical syndrome but lacking audiometric confirmation of hearing loss are classified as probable MD (2).

The underlying pathophysiology of MD is attributed to endolymphatic hydrops, a pathological distension of the endolymphatic system within the inner ear (3). While this disease is highly prevalent in the human literature, reports of potential MD equivalents in veterinary medicine remain limited. In geriatric dogs, peripheral vestibular syndrome is a common clinical presentation (4, 5). The established differential diagnoses for canine peripheral vestibular disorders include idiopathic vestibular syndrome (IVS), otitis media or interna, neoplasia, hypothyroidism, and ototoxicity (6). Furthermore, several functional, non-structural disorders have been proposed as the underlying etiology of IVS, which may include benign paroxysmal positional vertigo, vestibular neuritis, vestibular migraine, and vestibular seizures (7, 8).

The standard diagnostic work-up for a peripheral vestibular syndrome in dogs typically includes a clinical and neurological examination, serum biochemistry, serum total T4 levels, blood pressure measurement, otoscopy, and magnetic resonance imaging (MRI) of the head (7). Although auditory brainstem response (ABR) testing provides a valuable functional assessment of the cochlear pathway, it is not routinely incorporated into standard veterinary clinical practice. Consequently, fluctuating or progressive hearing loss in veterinary patients presenting with episodic vestibular signs often remains undetected.

This case report describes the clinical presentation, diagnostic findings, and long-term clinical course of a dog with recurrent peripheral vestibular episodes. In the absence of structural abnormalities on MRI, functional auditory testing via ABR and distortion-product otoacoustic emissions (DPOAE) documented sensorineural hearing impairment. These findings parallel, to some degree, the clinical and diagnostic criteria of Ménière’s disease in humans, suggesting an MD-like disease in dogs.

## 2 Case description

*Initial presentation*: In 2022, a 15-year-old mixed-breed male dog (15 kg) presented with three brief, self-resolving vestibular episodes over the past 2 weeks. Lasting 30–60 min, these attacks involved distress, disequilibrium, recumbency, falling to the right, trembling, panting, right-sided head tilt, and rapid nystagmus (direction unknown). The owner also reported a recent worsening of hearing impairment. Between episodes, the owners reported that the dog appeared unremarkable in terms of head posture, eye movements, and walking.

At presentation, a systolic heart murmur (3/6) was noted, consistent with known mitral and tricuspid valve insufficiency (both CHIEF stage B2) (9). Prior to presentation, the dog was already receiving treatment prescribed by the referring veterinarian, consisting of pimobendan (0.3 mg/kg in the morning and 0.15 mg/kg in the evening) and propentofylline (3 mg/kg BID). Clinical examination and body condition score (5/9) were otherwise unremarkable, reflecting a history of good general health.

*Hearing questionnaire and otoscopy*: The owner reported that the dog’s hearing had noticeably decreased since the onset of the episodes. A hearing questionnaire completed by the owner yielded a score of 8/8 on the scale (0 = normal, 8 severe hearing impairment), consistent with severe bilateral hearing impairment (10).

Otoscopy revealed mild bilateral otitis externa. The left ear canal had moderate-to-marked cerumen occluding the tympanic membrane. Cytology revealed occasional cocci. The right ear canal contained mild cerumen, and the tympanic membrane was intact. Cytology showed a small amount of Malassezia sp. (1+). Otitis scores were 5/12 (right) and 4/12 (left) (11).

*Neurological examination*: The dog presented with normal mentation and a mild right-sided head tilt. The gait was normal at that time, and ataxia was no longer present. Cranial nerve examination showed a positional ventral strabismus of the right eye, and positional rotatory nystagmus on head and neck extension (direction unknown). Postural reactions and spinal reflexes were unremarkable. There was no pain on palpation of the spine or head. The clinical findings were consistent with a right-sided peripheral vestibular disease.

*Diagnostic investigations*: Non-invasive oscillometric measurement of arterial blood pressure was 156/77 mmHg (MAP 106 mmHg, mean of five measurements). Complete blood count, blood gas, serum biochemistry, and an Angiostrongylus vasorum snap test (IDEXX Laboratories, Kornwestheim) were unremarkable. Thromboelastography indicated hypercoagulability (MA 79 mm ↑, reference: MA: 42.9–67.9) with normal R, K, Angle, and LY30 values.

Abdominal ultrasonography revealed only moderate splenomegaly with otherwise unremarkable findings. Thoracic radiography in two views (ventrodorsal and laterolateral) demonstrated no evidence of pulmonary or mediastinal neoplasia but showed cardiomegaly (vertebral heart score 11.5, reference range: 8.5–10.5).

**Figure 1:**
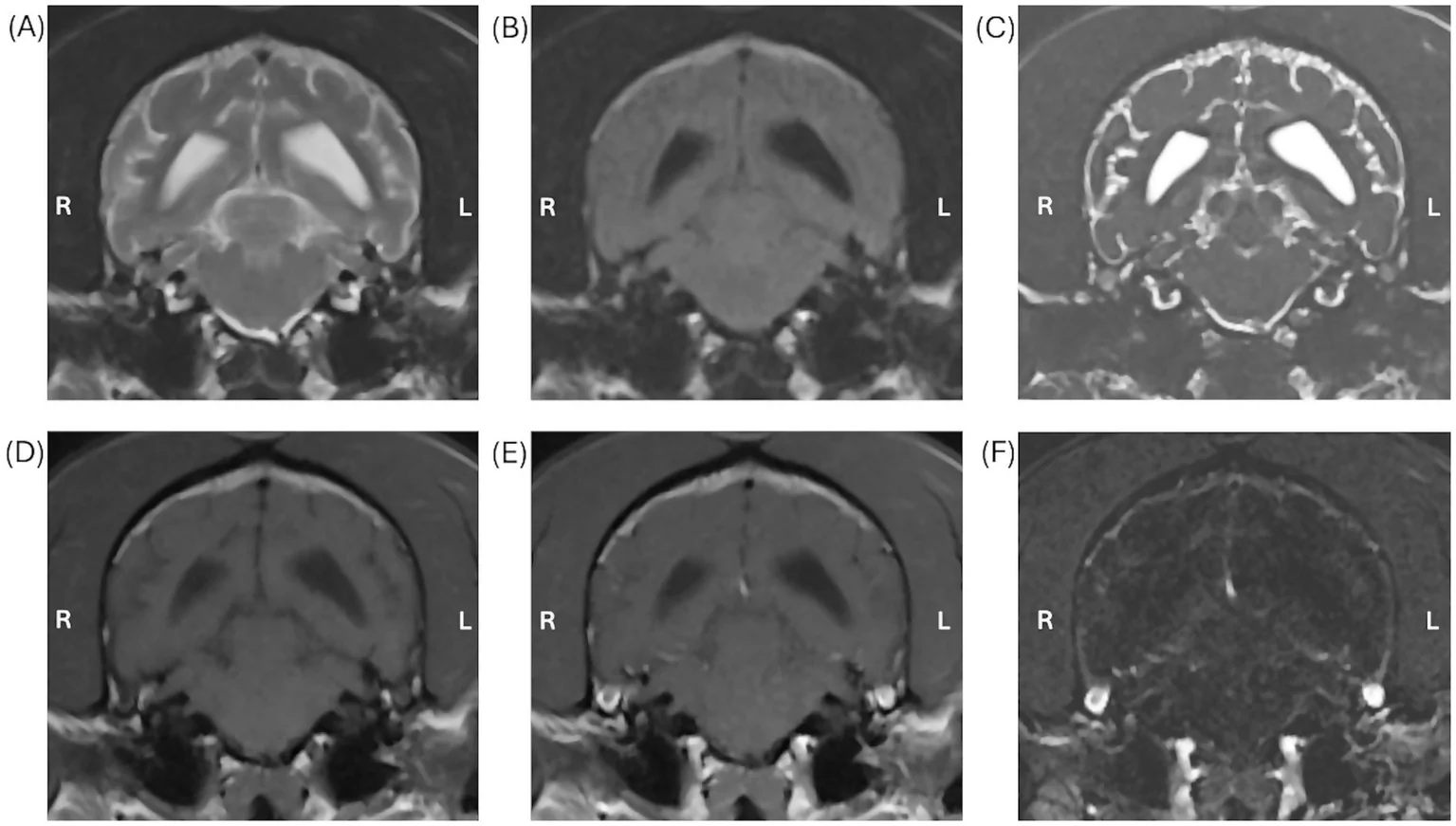
Multimodal transverse MRI of the head and inner ear structures. **(A)** T2-weighted (T2W) and **(B)** Fluid-Attenuated Inversion Recovery (FLAIR) sequences show the physiological signal intensity of the intra-labyrinthine fluids in T2W **(A)** and complete signal suppression on FLAIR **(B)**. **(C)** High-resolution CISS (Constructive Interference in Steady State) sequence depicting bilaterally symmetrical nerve complex of the vestibulocochlear (CN VIII) and facial (CN VII) nerves with no volume increase or change in signal intensity. **(D–F)** T1-weighted pre-contrast **(D)**, post-contrast **(E)**, and subtraction **(F)** images show no pathological contrast enhancement.

**Figure 2:**
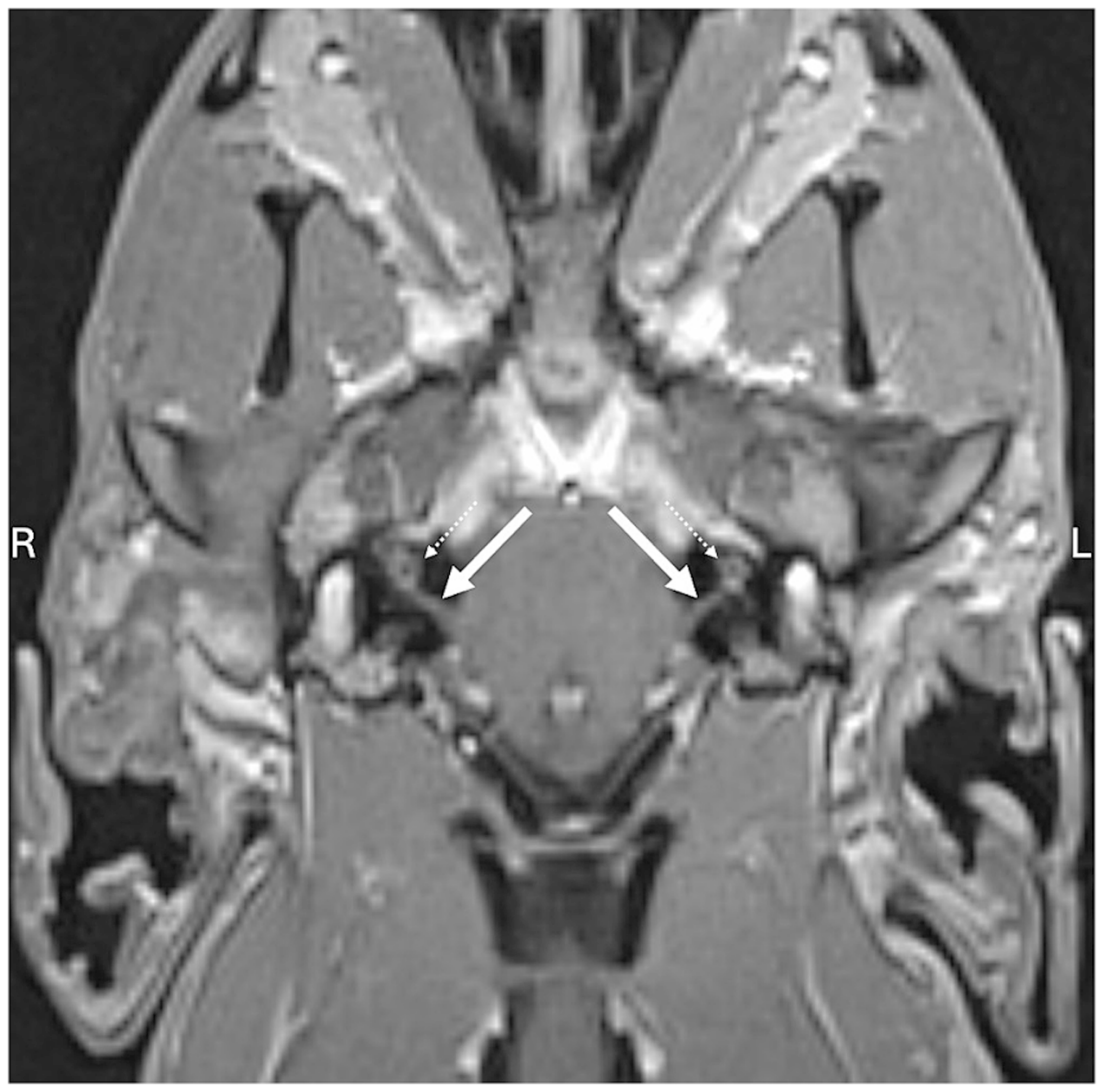
Dorsal multiplanar reconstruction (MPR) of a T1-weighted MRI of the dog. Solid arrows indicate the common trunk of the Vestibulocochlear nerve (CN VIII), which subsequently divides into the vestibular and cochlear nerves, and the Facial nerve (CN VII) as they emerge from the brainstem and enter the petrous part of the temporal bone. No structural abnormalities are identified within the highlighted neural or the adjacent soft tissue structures. The dashed arrows point to the geniculate ganglion, located at the external genu of the facial canal.

*MRI and CSF analysis*: Anesthesia was maintained with isoflurane following butorphanol (0.2 mg/kg IV) and propofol induction. For magnetic resonance imaging (T2W, FLAIR, T2W*, CISS, DW, T1W pre and post contrast, MPR pre and post contrast, field of view: 256 × 256 mm, slice thickness for all sequences 3 mm, CISS and MPR 0.8 mm, with ear protection, 1.5-T MRI scanner [Magnetom Symphony, Siemens Medical Systems, Erlangen, Germany)] intravenous contrast medium Gadoteric acid (Dotavision®, 0.5 mmol/mL) was administered intravenously at 0.15 mmol/kg (0.3 mL/kg). It revealed unremarkable middle and inner ear structures, with a normal appearance of the intra-labyrinthine fluid signal on T2W sequences and complete suppression on FLAIR sequences. The thalamus, cerebellum, brainstem, and cranial nerves demonstrated normal morphology and signal intensity throughout. Few cerebral microbleeds on T2* in the right frontal and parietal lobes, and mild ventriculomegaly, were considered age-related changes and clinically insignificant. No pathologic contrast enhancement was observed. Specifically, the midbrain, pons, and medulla oblongata were unremarkable, with no evidence of focal lesions, signal abnormalities, or mass effect. The common trunk of the vestibulocochlear and facial nerves was identified on T2W and was symmetrical, not enlarged, and showed no abnormal contrast enhancement on post-contrast T1W, MPR, and subtraction (Figures 1, 2). High-resolution T2W CISS imaging demonstrated normal fluid signal within the endolymphatic and perilymphatic spaces of the bilateral cochleae (Figure 2). The tympanic bullae appeared unremarkable, with no evidence of effusion or inflammatory changes suggestive of otitis media. The external auditory canals demonstrated slight heterogeneity in their deeper portions with minimal contrast enhancement at the periphery, findings suggestive of a mild inflammatory reaction consistent with otitis externa.

A cisternal puncture was performed at the atlanto-occipital space for the collection of cerebrospinal fluid. The analysis showed an unremarkable total nucleated cell count (leukocytes: 0/μl, erythrocytes: 1/μl), microprotein (0.24 g/L), and lactate (2.48 mmoL/L), and unremarkable cytology on cytospin preparations.

*Hearing testing*: After the imaging and CSF tap, the dog underwent hearing testing under anesthesia. Comprehensive hearing testing used a mobile device (Cubaudio®, PathMedical GmbH, Germering, Germany; Article No. 100360-CUB, distributed for veterinary use by Dr. Ing. Hans Oswald Ingenieurdienstleistungen, Oberpframmern, Germany; www.oexing.de/), initially designed for newborn hearing screening.

Ears were cleaned with a dry cotton swab to remove any leftover cerumen. Two different hearing test modalities were used. First, the rapid auditory brainstem response (ABR) test was used to determine the hearing threshold with broadband click stimuli, as previously reported by Stanger et al. (12). Insert earphones and three dermal stainless-steel needle electrodes were used (12 × 0.40 mm; Natus Europe GmbH, Planegg, Germany). Needles were placed SC at the tragus of the right ear and connected to the cathode (negative, inverting electrode), at the vertex at position Cz connected to the anode (positive, non-inverting electrode), and in the neck (ground). Bilateral auditory assessment utilized an interleaved stimulus protocol (PATH medical system) with clicks delivered alternately to each ear with a precise time offset. This sequential stimulation enabled simultaneous ipsilateral (red, right ear) and contralateral (blue, left ear) ABR recordings. The stimulation intensities decreased in 10 dB steps from 90 dB nHL to 0 dB nHL, and the system varied the averaging based on the recognition of the peak V, ranging from a few to 2000 averaged sweeps. The lowest stimulation intensity with an identifiable peak V on the ABR recording was defined as the hearing threshold. This protocol measured the click hearing threshold in 2 min.

The second modality involved distortion-product otoacoustic emissions (DPOAEs) to evaluate frequency-specific hearing. Stimulus pairs with L1 = 65 dB SPL and L2 = 55 dB SPL were used to test eight frequencies. DPOAEs were recorded using stimulus tone pairs with f2 primary frequencies 1, 1.5, 2, 3, 4, 5, 6, and 8 kHz and a constant f2/f1 frequency ratio of 1.22. Correct probe placement and acoustic sealing were verified via the system’s quality control using disposable memory foam ear tips (PATH Medical, Germering, Germany). Measurements were conducted in a quiet, noise-shielded environment.

**Figure 3:**
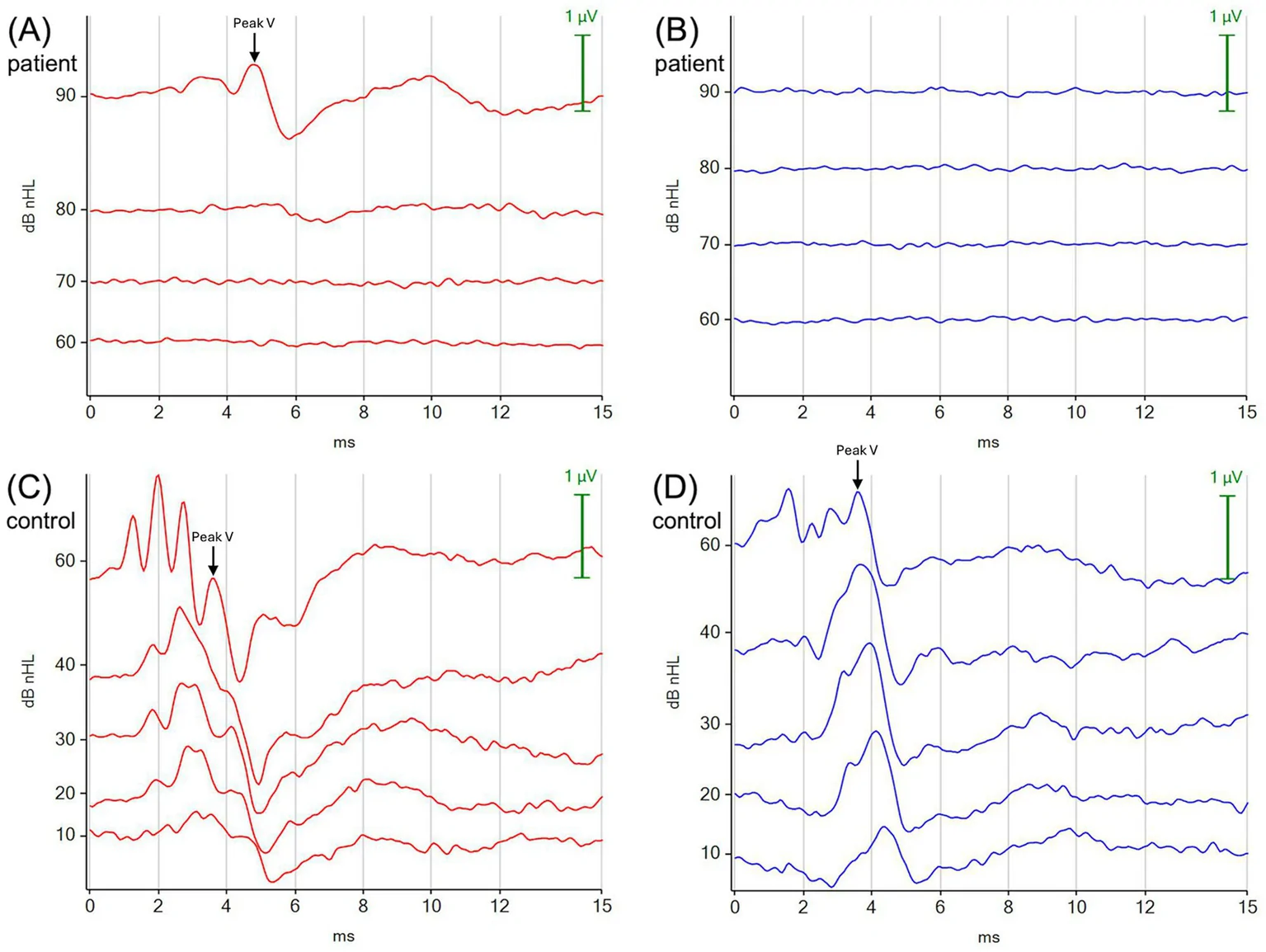
Auditory brainstem response (ABR) with a rapid hearing screening protocol in the patient **(A, B)** and a normal-hearing control dog **(C, D)**. Measurements were performed using bilateral sequential stimulation with broadband clicks at a stimulus rate of 30 Hz with intensities decreasing stepwise. The device dynamically adjusted the averaging procedure when peak V was clearly identified (ranging from a few to 2000 sweeps). The recording electrode was placed at the tragus of the right ear. The lowest stimulus intensity at which peak V was visible was defined as the hearing threshold. Patient: **(A)** Right ear (ipsilateral recording): Hearing threshold 80 dB nHL, **(B)** Left ear (contralateral recording): No peak V up to 90 dB nHL, hearing threshold > 90 dB nHL. Control dog: **(C)** Right ear, **(D)** Left ear: Hearing threshold 10 dB nHL.

**Figure 4:**
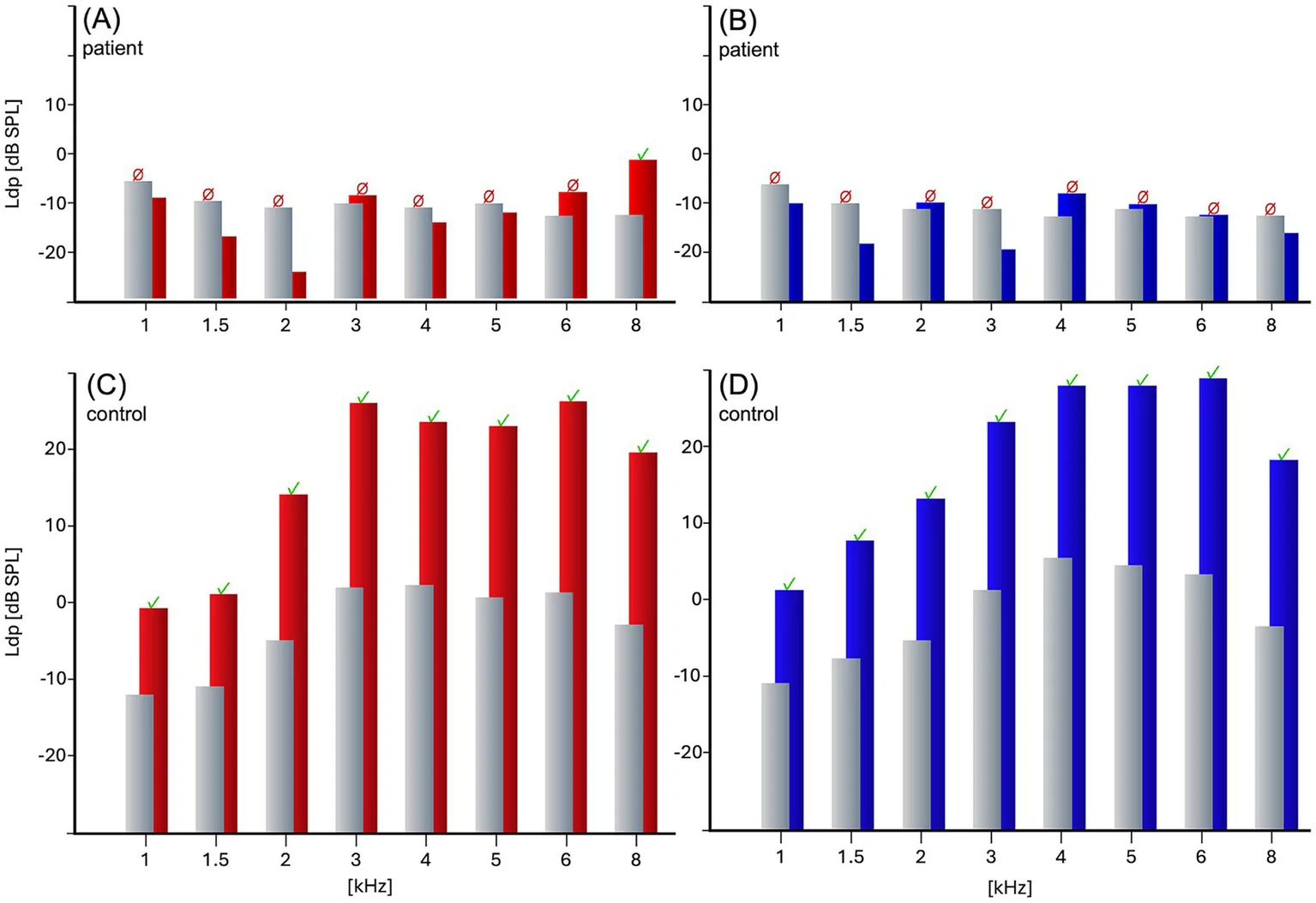
DPOAE audiograms (L1/L2 = 65/55 dB SPL; 1–8 kHz) from the patient **(A, B)** and a normal-hearing control dog **(C, D)**. DPOAEs were recorded after a quality control check, with a probe tightly inserted into the external ear canal. Colored bars represent the DPOAE amplitude in dB SPL (Ldp, level of distortion product), and grey bars the corresponding noise floor in dB SPL. Valid emissions, defined by a signal-to-noise ratio ≥ 6 dB, are marked with a green checkmark. Patient: **(A)** Right ear (red): DPOAE were absent up to 6 kHz and recorded at 8 kHz, **(B)** Left ear (blue): DPOAE were absent across all tested frequencies. Control dog: **(C)** Right ear (red) and **(D)** Left ear (blue), demonstrating robust, physiological DPOAE responses across the entire frequency spectrum (1–8 kHz).

*Results*: The click ABR hearing thresholds indicated severe hearing impairment in the right ear (click hearing threshold 80 dB nHL), and deafness with no detectable ABR in the left ear (click hearing threshold > 90 dB nHL), as shown in Figure 3. The DPOAE responses were detected only at 8 kHz in the right ear and were absent at the lower tested frequencies; in the left ear, DPOAE responses were absent at all tested frequencies, as illustrated in Figure 4.

*Treatment*: Supportive treatment consisted of intravenous fluids, maropitant (1 mg/kg PO q24; Cerenia, Zoetis, Parsippany, NJ, USA), and clopidogrel (1.25 mg/kg PO q24h; Clopidogrel 1 A Pharma, 1A Pharma GmbH, Oberhaching, Germany); propentofylline administration was discontinued. The owner reported a gradual improvement of the mild head tilt over 1 month. A repeat neurologic examination performed 4 weeks later was normal.

*Follow-up and long-term outcome*: In total, the dog experienced six episodes of acute vestibular signs between 2022 and 2025, up to the age of 18 years.

The initial three episodes occurred in April 2022, were brief, and resolved within 1 month. Following gradual improvement, the dog remained clinically unremarkable, with no vestibular signs. After a 25-month symptom-free interval, a fourth vestibular attack occurred in May 2024. During this recurrence, the dog presented with a right-sided head tilt, ipsilateral falling, and bilateral positional rotatory nystagmus on head and neck extension; these signs resolved spontaneously with symptomatic care.

After another 12 months of clinical remission, two additional episodes were recorded in May 2025. One of these was a severe attack lasting approximately 45 min. The dog was recumbent, unable to stand, and exhibited bilateral positional rotatory nystagmus on head and neck extension during this episode.

Overall, the dog returned to almost its normal neurological status, except for a persistent mild right-sided head tilt and a consistent hearing impairment. Despite these recurrent attacks, the dog maintained a good quality of life until 18 years of age. Further long-term development beyond this period is currently unavailable.

## 3 Discussion and conclusion

Over 3 years, the dog had three episodes of recurrent vestibular signs, totaling six individual vestibular attacks, each lasting 30 min to a few hours. The owners reported a recent worsening of hearing impairment, at the same time as the first attacks. Standard imaging of the head and ear failed to identify any structural changes in the neuroparenchyma, middle ear, or inner ear that could explain the episodes or the hearing loss.

Ménière’s disease is clinically defined by recurrent episodes of spontaneous vertigo, accompanied by fluctuating sensorineural hearing loss primarily affecting low- to mid-frequency sounds (<2000 Hz) (13), tinnitus, and a sense of aural fullness (2). While this audiometric pattern is typical for early-stage human MD, the audiogram tends to flatten as the disease progresses to advanced stages, reflecting significant and permanent hearing impairment comparable to that of this dog (14, 15). While most patients present with unilateral symptoms, bilateral MD occurs in a subset of patients and is generally associated with a more severe and progressive clinical course (16).

The dog’s presentation, marked by time-matched onset of recurrent vestibular attacks and progressive hearing impairment, aligns with the clinical signs of MD. Consistent with human MD, where standard MRI of the inner ear is usually unremarkable, advanced imaging in this dog revealed no structural lesions. Delayed post-gadolinium high-resolution 3D-FLAIR MRI can visualize endolymphatic hydrops (17). This finding is considered the pathological hallmark of MD in most cases.

Hydrops-induced membrane ruptures are thought to mix potassium-rich endolymph with perilymph, disrupting the potassium gradient necessary for hair cell excitability and triggering acute vestibulocochlear signs (18). Proposed etiologies include endolymph dysregulation, autoimmune reactions, ionic imbalances, or vascular factors (3, 18, 19).

Visualization of the hydrops has become a valuable tool for distinguishing MD from other vestibular disorders, such as vestibular migraine (20).

In the present case, sensorineural hearing loss was documented via ABR and DPOAE, confirming cochlear involvement. Click ABR testing revealed no detectable responses in the left ear (click hearing threshold > 90 dB nHL), consistent with unilateral deafness. In the right ear, only residual hearing was preserved (click hearing threshold 80 dB nHL), with residual high-frequency cochlear function indicated by 8 kHz DPOAE responses. When interpreting these electrodiagnostic findings, potential artifacts must be considered. Notably, as shown in cats, high-volume ABR stimuli can trigger vestibular-evoked potentials that can mimic auditory responses (21). A similar phenomenon may also occur in dogs; if this were the case here, the actual hearing loss might be even more profound than recorded. Although environmental or respiratory noise can interfere with low-amplitude DPOAEs, strict internal quality control with an acoustically sealed probe was maintained in clean, unobstructed ear canals. Consequently, the absent DPOAEs, combined with a lack of middle-ear pathology, confirm genuine cochlear hair-cell dysfunction and sensorineural hearing loss rather than a conductive artifact. While DPOAEs assess peripheral hair cell function, ABR evaluates the retrocochlear auditory pathway within the brainstem. The preferential loss of low- and middle-frequency DPOAE emissions, with a preserved measurable response at 8 kHz in the right ear, parallels the audiometric findings seen in human MD. A limitation of this study is that this frequency pattern was not further corroborated using tonal ABR or ASSR (22).

Additionally, given the patient’s geriatric status, age-related presbycusis remains a differential diagnosis, as dogs over 10–12 years of age commonly exhibit high-frequency hearing loss due to outer hair cell degeneration (23). However, presbycusis typically targets high frequencies first, whereas this dog maintained residual function at 8 kHz. Furthermore, the owner noted a recent worsening of hearing that closely matched the initial vestibular episode, which is a characteristic of MD. This distinct clinical pattern and severity suggest a pathological process beyond physiological aging, supporting an MD-like disorder and arguing against benign paroxysmal positional vertigo (BPPV), which is a condition frequently discussed in geriatric idiopathic vestibular syndrome. Nevertheless, a preexisting component of presbycusis cannot be entirely excluded.

The diagnostic workup of the presented dog ruled out numerous causes of structural brain and ear disease. However, the clinical significance of the microbleeds observed on MRI remains uncertain; MRI review failed to identify microbleeds in the brainstem and thalamus, e.g., in locations previously discussed as possible origins of recurrent vestibular attacks in two dogs (24). Yet, microvascular pathologies affecting the inner ear cannot be definitely ruled out (25, 26). Systolic blood pressure (156/77 mmHg, MAP 106 mmHg) was classified as prehypertensive with low risk of target organ damage per ACVIM guidelines (27). As recommended, regular monitoring over 3 years revealed no changes in blood pressure, renal function, or vision.

Other differential diagnoses for peripheral vestibular syndrome in geriatric dogs include idiopathic vestibular syndrome (IVS), hypothyroidism, and ototoxicity. Although IVS is prevalent in older dogs and can relapse in up to 60% (28), its signs typically resolve over days to weeks. In contrast, this dog’s brief, recurrent episodes lasting minutes to hours are uncharacteristic of IVS (7) but typical for MD (2). Evaluation of hearing impairment in dogs with IVS could help identify parallels with MD, thereby differentiating it from BPPV, a frequently discussed etiology of IVS (7).

Concurrently, hypothyroidism can induce cranial polyneuropathies affecting the vestibulocochlear (CN VIII) nerve (29) or ischemic strokes resulting in central signs (30). However, normal thyroid testing ruled out this etiology.

Ototoxicity was also considered, as systemic aminoglycosides or topical medications breaching a compromised tympanic membrane can exert potent vestibulotoxic and cochleotoxic effects, resulting in peripheral vestibular signs and sensorineural deafness (31, 32). This differential was excluded based on a negative history of ototoxic medication, absent ear-cleaning procedures, and the episodic clinical signs, which contrasts with the acute onset and static or resolving course typical of toxic insults.

Furthermore, while vestibular epilepsy has been proposed in veterinary medicine as a cause of paroxysmal vestibular signs without EEG confirmation, this central syndrome does not account for the residual right-sided head tilt, the concurrent, progressive sensorineural hearing impairment, and the cochlear outer hair cell dysfunction documented in this dog, making a primary epileptic etiology highly improbable (8). However, since an EEG was not performed in this patient, a focal epileptic origin cannot be fully ruled out, which represents a limitation of this case.

Finally, congenital disorders, trauma, and neoplasia were ruled out based on the patient’s age, history, and unremarkable advanced imaging findings (6).

The case presented here is clinically significant, as spontaneous MD is nearly unreported in the veterinary literature. There is a paucity of peer-reviewed case reports describing dogs with clinical features resembling MD. One abstract described a young dog with recurrent peripheral vestibular episodes (termed “vestibular paroxysm”) that was attributed to an atypical MD-like disorder. However, unlike the present case, that dog did not exhibit hearing loss, and the diagnosis remained presumptive without advanced hearing testing (33). To the authors’ knowledge, this is the first case report providing comprehensive auditory evidence of an MD-like syndrome in a dog. This report draws a parallel between human and canine vestibulocochlear disorders, raising the question of whether MD-like clinical features occur more frequently in dogs than previously recognized. This case may be one of the first to meet key diagnostic criteria for MD. It demonstrates a triad of symptoms: episodic vestibular dysfunction, progressive sensorineural hearing loss, and the exclusion of other possible etiologies. A definitive confirmation of an animal model for MD would require further examinations, such as specialized MRI techniques to verify endolymphatic hydrops. Advanced hearing testing in dogs with vestibular disease is indicated to determine whether vestibular episodes and hearing loss occur concurrently, and consequently, whether an MD-like syndrome exists in a subset of dogs with peripheral vestibular disease.

## Data Availability

The original contributions presented in the study are included in the article/supplementary material, further inquiries can be directed to the corresponding author.
